# Binge drinking in Brazil during the COVID-19 pandemic: why does gender still matter?

**DOI:** 10.47626/1516-4446-2025-4240

**Published:** 2025-12-18

**Authors:** Maria A.T. Ribeiro, Matheus B. Costanzi, Andrea F. Mello, Marcelo F. Mello, Vitor C. Calegaro, Thiago M. Fidalgo

**Affiliations:** 1 Departamento de Psiquiatria, Universidade Federal de São Paulo, São Paulo, SP, Brazil; 2 Universidade Federal de Santa Maria (UFSM), Santa Maria, RS, Brazil; 3 Departamento de Neuropsiquiatria, UFSM, Santa Maria, RS, Brazil

**Keywords:** Behavior, drinking, gender roles, pandemic

## Abstract

**Objective:**

The COVID-19 pandemic affected public health worldwide. Binge drinking is a high-risk behavior pattern linked to health problems and injuries. We aimed to assess the prevalence of binge drinking at four time points during the 1st year of the pandemic and correlate these with sociodemographic variables, providing insights into binge drinking trends.

**Methods:**

This cross-sectional study analyzed data from a national survey conducted between April 2020 and January 2021 with 11,205 adult participants. Sociodemographic variables included sex, age, sexual orientation, race, education level, and health care worker status. Regression models were used to assess associations with past-month binge drinking, which was the dependent variable.

**Results:**

Binge drinking rates were stable across the four time points. Male sex was consistently associated with binge drinking at all time-points. Other variables such as younger age, lower educational background, and non-straight sexual orientation were associated with binge drinking at some, but not all, time points.

**Conclusion:**

The persistence of gender disparities in binge drinking during a period of high unprecedented stress suggests the need for targeted interventions in future public health emergencies. Further research is required to explore the pandemic’s long-term effects on alcohol consumption behaviors and related health outcomes.

## Introduction

The first known COVID-19 death in Brazil occurred in March 2020.^1^ At that point, the disease’s potential to cause death among older adults, adults, and even children led government officials to turn their attention to containing the spread of the virus. Based on observations from other pandemics, a drastic psychosocial impact that would outlast the outbreak was expected, as was a significant increase in mental health symptoms and changes in substance use patterns.^2^,^3^ Significant psychosocial stressors, such as unemployment and working exclusively from home, may have led to changes in alcohol consumption patterns.

Other studies have investigated drinking patterns during the COVID-19 pandemic. In a European sample, Kilian et al.^4^ found that alcohol use decreased during the initial months of the pandemic, hypothesizing that lower availability could have been a factor. Kilian et al.^5^ conducted a meta-analysis of observational studies from similar samples and found that drinking decreased more often than it increased.

Alcohol consumption is an essential risk factor for many diseases, such as liver disease, hypertension, cancer, and hemorrhagic stroke.^6^ It is also one of the three leading risk factors for global disease burden.^7^ Higher alcohol consumption levels are more associated with all-cause mortality risk, beginning at lower consumption levels in women than men.^8^ Alcohol intake can also aggravate symptoms of depression.^9^ According to Nutt et al.,^10^ alcohol is the most harmful drug to others, given the increased risk of injury, crime, and overall harm to the community, scoring higher than heroin or crack cocaine.

Binge alcohol consumption (heavy episodic drinking), an indicator of harmful use, is associated with an elevated potential for toxicity^11^ and may be associated with other negative consequences, such as unsafe sexual behavior, sexually transmitted infections,^12^,^13^ and neurocognitive impairment, especially in adolescents.^14^ Naimi et al.^15^ found that binge drinkers are 14 times more likely to drive while impaired than non-binge drinkers.

The COVID-19 pandemic had a significant effect on all types of violence against women, especially domestic violence. This has been called “the shadow pandemic.”^16^ Aggressive behavior is commonly associated with alcohol use and psychiatric disorders.^17^ Binge drinking is strongly linked to violence and injuries^18^ and could also have affected domestic violence during social isolation. Chronic alcohol consumption may also affect immune response to SARS-CoV through aberrant inflammation and reduced antiviral response in the lungs.^19^

This study aimed to assess the prevalence of binge drinking behavior at four time points during the 1st year of the pandemic and correlate the prevalence data with various sociodemographic variables, providing a comprehensive understanding of binge drinking patterns during this unprecedented period.

## Methods

Data were collected through a web-based survey on the SurveyMonkey platform. An electronic survey was selected due to the possibility of reaching more participants without violating social isolation measures in Brazil. To maximize the sample, we used a structured outreach strategy, including social media (Facebook, Instagram, Twitter, and LinkedIn), corporate mailing lists (targeted to higher education institutions, government agencies, and professional councils), press media, broadcast news on radio and television, and popular messaging applications in Brazil (WhatsApp and Messenger). The outreach campaign reported that the study was anonymous and that consent was required to participate. This communication strategy was continuous, including weekly live YouTube transmissions. The strategies were reinforced two weeks before each stage of the study. All participants received up to three e-mail messages reminding them to fill out the next questionnaire. Some paid boosting was directed to the northern, northeastern, and midwestern regions of the country, which were underrepresented.

Data were collected at four time points: T0 – 2 weeks in April and May 2020 (n=3,625), T1 – June 2020 (n=3,435), T2 – September and 2 weeks in October 2020 (n=2,303), and T3 – January 2021 (n=2,238). A complete description of this study’s methodology is available elsewhere.^20^

We used validated questionnaires to assess anxiety and depressive symptoms, trauma, sleep disturbances, and sociodemographic information. We also included a question concerning binge drinking (“How often do you drink six or more alcoholic drinks at one sitting?”). Those who did not answer this question were excluded from the analysis, which left us with 3,388 participants at T0, 2,775 at T1, 2,027 at T2, and 1,871 at T3. The questionnaire required about 20 minutes to fill out.

### Variables

The outcome of interest was binge drinking. Those who reported a monthly or greater frequency of binge drinking were considered positive. The control variables were sex (male or female), race (White or non-White), educational background (≤ 9 years or > 9 years), age (18-39 years or ≥ 40 years), sexual orientation (straight or non-straight), and employment in the health care sector (medical doctors, nurses, physical therapists, psychologists, etc. – yes or no).

### Data analysis

Extensive data processing and preparation were performed. Patients with invalid email addresses were excluded from the study. To identify possible duplicates, a rigorous process was conducted: 1) username and domain separation; 2) correcting usernames and domains by removing special characters, empty spaces, and by replacing incorrect domains (typing errors); 3) screening for potential duplicates through similar usernames in different domains; 4) manual verification of duplicates (sex, age, city, education, profession, etc.); and 5) merging duplicates and maintaining single confirmed cases.

First, we conducted a descriptive analysis to identify which variables were associated with binge drinking at T0, T1, T2, and T3. The covariables sex, race, educational background, age, sexual orientation, and health care employment were tested. All variables were associated with binge drinking at one or more time points (p < 0.20). We then conducted four weighted logistic regression models (T0, T1, T2, and T3), with binge drinking as the dependent variable in each model. Sex, race, educational background, age, sexual orientation, and employment in the health care sector were independent variables. We obtained odds ratios from the logistic regression models, which tested associations between binge drinking and the independent variables using the logistic command in Stata. A 5% significance level was used in all statistical analyses, which were performed in IBM SPSS Statistics 23 and Stata 13.

### Ethics statement

The data were stored on the SurveyMonkey server and were accessed through the lead investigator’s account. The database was anonymized, de-identified, and can be accessed upon request to the lead investigator (VC). This study was approved by the National Research Ethics Commission (Comissão Nacional de Ética em Pesquisa [CONEP]: decision 23081.019155/2020-14).

## Results

**Figure 1:**
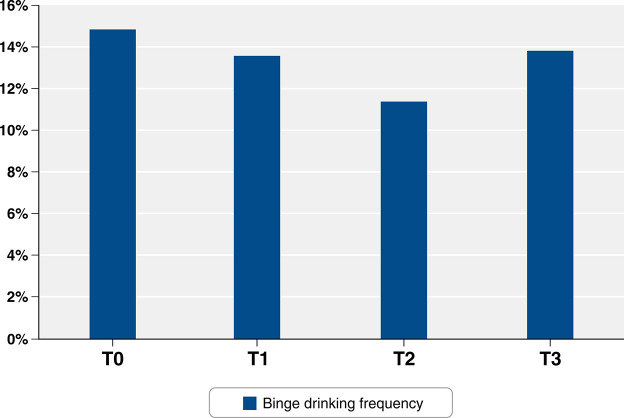
Binge drinking frequency.

At T0, our sample consisted of 3,388 participants, of whom 14.84% reported binge drinking at least once a month. Compared to the non-binge drinking group, this group had a higher prevalence of participants who were male, non-White, less educated, under 40 years of age, non-straight, and were not employed in the health care sector. At T1, 13.55% of the sample (n=2,775) reported binge drinking, and this group also had a higher prevalence of participants who were male, non-White, less educated, under 40 years of age, and non-straight than the non-binge drinking group. At T2, 11.35% of the participants (n=2,027) reported binge drinking, and this group had a higher prevalence of participants who were male, less educated, under 40 years of age, and non-straight, than the non-binge drinking group. At T3, 13.79% of the sample (n=1,871) reported binge drinking, and this group had significantly more participants who were male, less educated, under 40, and non-straight than the non-binge drinking group. More detailed information is presented in Table 1 and Figure 1.

In the adjusted logistic regression model, male sex, lower educational background, younger age (< 40), and non-straight sexual orientation were independently associated with binge drinking at T0 and T1 (Table 2). At T2, male sex and younger age (< 40) were independently associated with binge drinking. Finally, at T3, male sex and lower educational background were independently associated with binge drinking.

## Discussion

In this sample, binge drinking was stable over time: 14.84% at T0, 13.55% at T1, 11.35% at T2, and 13.79% at T3. Binge drinking was consistently associated with male sex. Non-straight sexual orientation was also independently associated with binge drinking at T0 and T1, but not at the other two time points.

The prevalence of binge drinking in our sample was similar to that found in a large Brazilian populational study prior to the COVID-19 pandemic, i.e., 16.5% in the last 30 days. Sex differences in binge drinking were also significant: the prevalence was 24% among men and 9.5% among women.^21^ This difference is similar to our results, in which the rate of binge drinking among men was more than double that of women at each time point. During the COVID-19 pandemic, a cross-sectional Brazilian study found that 68.9% of the participants consumed alcohol during social isolation, of whom 22.7% reported increased consumption and 32.5% decreased consumption compared to the pre-pandemic period.^22^ That study, however, did not inquire about binge drinking.

In Mexico, another cross-sectional survey conducted at the beginning of the COVID-19 pandemic found that 14% of the respondents engaged in binge drinking, a proportion similar to our findings. They also found that binge drinking was more prevalent in participants who were not in lockdown (20%) than in those who were (12%).^23^ Villanueva-Blasco et al.^24^,^25^ found a decreased frequency of binge drinking among both men and women during the COVID-19 lockdown in Spain, but the rate was more pronounced among men. It is important to emphasize that, due to methodological differences, these studies are not comparable with our study or among themselves.

Gender disparities in drinking patterns have been consistently reported worldwide.^26^ Critical biological differences in men’s and women’s alcohol metabolism^27^-^29^ have been considered part of the reason for this. Some differences involving cortisol and sexual hormones^30^,^31^ may also contribute to this difference. In addition, it has been shown that men and women have different relationships between stress and alcohol relapse,^32^,^33^ and women may suffer more significant adverse health consequences, even with lower levels of alcohol consumption.^34^-^36^ Messas et al.^37^ report that alcohol consumption may be a male role norm in Brazilian society. Alcohol consumption is not considered problematic in our culture, and a drinking problem is often only noticed after significant compromise to professional responsibilities. More recently, Radke et al.^38^ tested other mechanisms in mice, finding that sex differences in the nucleus accumbens and dopamine signaling could account for drinking differences between males and females.

This gap seems to be narrowing in recent decades.^39^-^42^ The prevalence of binge drinking is now similar between the sexes in many socioeconomically developed societies.^43^ This narrowing gap is especially significant among young adults, driven mainly by enhanced alcohol consumption among young women, as demonstrated in a meta-analysis by Slade et al.^39^ Cultural and psychological factors, such as women’s role in society^41^,^42^,^44^ and the adoption of male behavioral patterns^45^ may be contributing to this narrowing difference.

The reasons for the sustained gender gap in binge drinking in our sample during the COVID-19 pandemic remain unexplained. One study suggested that the coronavirus pandemic could have caused a reversion to traditional gender roles.^46^ It is suggested that critical existential threats (such as the pathogen) may make people more socially conservative. Similar effects were observed during the Ebola outbreak^47^,^48^ and after terrorist attacks.^49^ Schaller & Park^50^ hypothesized that the threat of infection could trigger a “behavioral immune system,” i.e., a set of behavioral changes and cognitive responses that could reinforce cultural differences and conformist attitudes in an effort to avoid infection.^51^,^52^ The coronavirus threat may have influenced conformity to gender roles, which could affect drinking patterns. Further research is needed to test this hypothesis.

It is important to note that non-straight sexual orientation was also independently associated with binge drinking in our study. Other studies have also found that this influence is significant in sexual minority women,^53^-^55^ and the same was observed in our study (data available upon request). Sexual minority stressors are related to cultural and social structures and can be associated with lower quality of life and higher alcohol- and drug-related problems among non-straight people. The reasons for increased binge drinking in sexual minority women may be nonadherence to traditional gender roles, including the adoption of more masculine traits (and drinking patterns) than heterosexual women. Among sexual minority men, greater concern with body image and more frequent social interaction with straight women (i.e., less pressure to drink heavily) may play a role in reduced binge drinking.^54^

These findings must be considered in the light of some limitations. We conducted a cross-sectional analysis of four non-comparable time points. Furthermore, this cannot be considered a longitudinal study because the sample varied over time. In addition, our sample is not representative of the Brazilian population, and the data should not be extrapolated. Caution is required when comparing our findings with pre-pandemic studies, as the role of this milestone event is still being comprehended. Finally, it is important to acknowledge that this study relied on self-reported alcohol consumptions patterns. According to Northcote et al.^56^ self-report measures are accurate regarding light or medium drinking episodes but could become less accurate when participants engage in heavy drinking due to underestimation. This could be especially relevant for this study, since our primary variable was binge drinking.

Overall, our findings suggest that binge drinking remained stable over four time points during the initial months of the COVID-19 pandemic (April 2020 to January 2021). The variable most consistently associated with binge drinking was male sex. Even though gender disparities in alcohol consumption have long been reported worldwide, we mode some hypotheses as to why this could have remained true at the beginning of the pandemic. We discussed the existential threat represented by the pandemic, which could have influenced drinking behavior and conformity to gender roles. Non-straight sexual orientation was also associated at some time points and was discussed separately. These findings suggest that the effects of the COVID-19 pandemic on alcohol consumption could require more time to appear than our study period. More studies are needed to address changes in alcohol consumption patterns over the following years – binge drinking is vital due to its harmful consequences for society and for individual health. Further research on this topic could identify groups that are vulnerable to binge drinking and could guide post-pandemic public policies regarding alcohol with a gender-sensitive approach. Longitudinal studies with representative samples of the Brazilian population should be conducted.

## Figures and Tables

**Table 1:** Descriptive statistics of the sample

	Binge T0 (freq. 14.82%)				Binge T1 (freq. 13.55%)				Binge T2 (freq. 11.35%)				Binge T3 (freq. 13.79%)			
	Yes (n=502)	No (n=2,886)	Total (n=3,388)	p-value	Yes (n=376)	No (n=2,399)	Total (n=2,775)	p-value	Yes (n=230)	No (n=1,797)	Total (n=2,027)	p-value	Yes (n=258)	No (n=1,613)	Total (n=1,871)	p-value
Sex				< **0.001**				< **0.001**				< **0.001**				< **0.001**
Male	190 (37.85)	613 (21.24)	803 (23.70)		139 (37.07)	485 (20.28)	624 (22.55)		95 (41.30)	361 (20.13)	456 (22.54)		87 (33.72)	320 (19.84)	407 (21.75)	
Female	312 (62.15)	2,273 (78.76)	2,585 (7.30)		236 (62.93)	1,907 (79.72)	2,143 (77.45)		135 (58.70)	1,432 (79.87)	1,567 (77.46)		171 (66.28)	1,293 (80.16)	1,464 (78.25)	
Race/ethnicity				**0.106**				**0.091**				**0.459**				**0.484**
White	420 (83.67)	2,493 (86.38)	2,913 (85.98)		296 (81.99)	1,996 (85.41)	2,292 (84.95)		190 (85.59)	1,540 (87.35)	1,730 (87.15)		223 (86.43)	1,419 (87.97)	1,642 (87.76)	
Non-White	82 (16.33)	393(13.62)	475 (14.02)		65 (18.01)	341 (14.59)	406 (15.05)		32 (14.41)	223 (12.65)	255 (12.85)		35 (13.57)	194 (12.03)	229 (12.24)	
Educational background (years)				< **0.001**				**0.003**				**0.072**				**0.012**
≤ 9	277 (55.18)	1,221 (42.31)	1,498 (44.21)		165 (47.41)	886 (38.95)	1,051 (40.07)		93 (40.79)	617 (34.74)	710 (35.43)		96 (37.21)	475 (29.45)	571 (30.52)	
> 9	225 (44.82)	1,665 (57.69)	1,890 (55.79)		183 (52.59)	1,389 (61.05)	1,572 (59.93)		135 (59.21)	1,159 (65.26)	1,294 (64.57)		162 (62.79)	1,138 (70.55)		
Age (years)				< **0.001**				**0.005**				**0.009**				**0.125**
< 40	411 (81.87)	2,050 (71.06)	2,461 (72.66)		273 (77.56)	1,613 (70.34)	1,886 (71.30)		176 (76.52)	1,217 (68.06)	1,393 (69.03)		180 (70.04)	1,013 (62.80)	1,193 (63.80)	
≥ 40	91 (18.13)	835 (28.94)	926 (72.66)		79 (22.44)	680 (29.66)	759 (28.70)		54 (23.48)	571 (31.94)	625 (30.97)		77 (29.96)	600 (37.20)	677 (36.20)	
Sexual orientation				< **0.001**				< **0.001**				**0.008**				**0.001**
Straight	415 (83.17)	2,578 (90.62)	2,993 (89.50)		277 (76.10)	1,990 (84.43)	2,267 (83.31)		159 (78.71)	1,368 (85.77)	1,527 (84.97)		200 (79.05)	1,373 (87.06)	1,573 (85.96)	
Non-straight	84 (16.83)	267 (9.38)	351 (10.50)		87 (23.90)	367 (15.57)	454 (16.69)		43 (21.29)	227 (14.23)	270 (15.03)		53 (20.95)	204 (12.94)	257 (14.04)	
Healthcare worker				**0.060**				**0.259**				**0.831**				**0.693**
No	438 (87.25)	2,423 (83.96)	2,861 (84.45)		326 (86.70)	2,026 (84.45)	2,,352 (84.76)		192 (83.48)	1,490 (82.92)	1,682 (82.98)		210 (81.40)	1,296 (80.35)	1,506 (80.49)	
Yes	64 (12.75)	463 16.04)	527 (15.55)		50 (13.30)	373 (15.55)	423 (15.24)		38 (16.52)	307 (17.08)	345 (17.02)		48 (18.60)	317 (19.65)	365 (19.51)	

Data presented as n (%) , unless otherwise specified.

T0 = 2 weeks in April and May 2020; T1 = June 2020; T2 = September and 2 weeks in October 2020; T3 = January 2021.

Bold type denotes statistical significance.

**Table 2:** Logistic regression

	Binge drinking at T0				Binge drinking at T1				Binge drinking at T2				Binge drinking at T3			
	cOR	aOR	95%CI	p-value	cOR	aOR	95%CI	p-value	cOR	aOR	95%CI	p-value	cOR	aOR	95%CI	p-value
Sex	0.442	0.428	0.349-0.526	< **0.001**	0.431	0.423	0.330-0.543	< **0.001**	0.358	0.343	0.250-0.470	< **0.001**	0.486	0.471	0.351-0.631	< **0.001**
Sexual orientation	1.954	1.744	1.326-2.292	< **0.001**	1.703	1.606	1.205-2.140	**0.001**	1.629	1.350	0.917-1.990	0.128	1.783	1.478	1.034-2.111	0.032
Race	1.238	1.137	0.870-1.486	0.346	1.285	1.223	0.899-1.663	0.199	1.163	1.116	0.722-1.726	0.619	1.148	1.022	0.677-1.542	0.916
Educational background	0.595	0.712	0.572-0.886	0.002	0.707	0.848	0.649-1.108	0.229	0.772	0.892	0.630-1.264	0.523	0.704	0.781	0.569-1.071	0.125
Age	0.543	0.626	0.483-0.811	< **0.001**	0.686	0.762	0.566-1.026	0.074	0.653	0.590	0.405-0.861	0.006	0.722	0.822	0.602-1.123	0.220
Health care worker	0.764	1.088	0.802-1.476	0.586	0.833	1.143	0.805-1.622	0.454	0.960	1.197	0.783-1.827	0.405	0.934	1.193	0.829-1.716	0.340

Bold type denotes statistical significance.

aOR = adjusted odds ratio; cOR = crude odds ratio; T0 = 2 weeks in April and May 2020; T1 = June 2020; T2 = September and 2 weeks in October 2020; T3 = January 2021.

## Data Availability

The data that support this study are available from the authors upon request.
